# Resolution Enhanced Array ECT Probe for Small Defects Inspection

**DOI:** 10.3390/s23042070

**Published:** 2023-02-12

**Authors:** Cai Long, Na Zhang, Xinchen Tao, Yu Tao, Chaofeng Ye

**Affiliations:** School of Information Science and Technology, ShanghaiTech University, Shanghai 201210, China

**Keywords:** array probe, eddy current testing (ECT), flexible sensor, small defects inspection, non-destructive testing

## Abstract

It is a continual and challenging problem to detect small defects in metallic structures for array eddy current testing (ECT) probes, which require the probe to have ultra-high resolution and sensitivity. However, the spatial resolution of an ECT array probe is limited by the size of the induction coils. Even if it is possible to increase the spatial resolution by using smaller coils, the sensitivity of the sensor also decreases. To obtain finer spatial resolution without sacrificing sensitivity, this paper proposes a resolution enhanced ECT array probe with four rows of coils attached to a flexible printed circuit board (FPCB). The distance between each two adjacent coils in a row is 2 mm and the position of each row is offset by 0.5 mm along the horizontal direction related to its prior row. The outputs of the four rows are aligned and interpolated in a line, and in this way the image resolution of the probe is increased to 0.5 mm. The probe is configured to operate with the differential setting, namely two differential coils operate simultaneously at each time. The currents in the two coils can be controlled to have the same flowing direction or opposite flowing direction, resulting in different distributions of the induced eddy current and two sets of output images. A patch-image model and an image fusion method based on discrete wavelet transforms are employed to suppress the noise and highlight the defects’ indications. Experimental results show that small defects with dimensions as small as length × width × depth = 1 mm × 0.1 mm × 0.3 mm on a 304 stainless-steel sample can be detected from the fused image, demonstrating that the probe has super sensitivity for small defects inspection.

## 1. Introduction

It is essential to inspect defects in critical industrial structures, such as nuclear power plants, transportation, aviation, petrochemical plants, etc. Non-destructive testing (NDT) technologies aim at evaluating materials/structures for characterizing differences and defects without causing damage to the objects under test [1,2]. The earlier a defect is identified, the less damage it can cause, which will save a lot of time and cost. However, the inspection of small defects on metal surfaces efficiently and economically is still a problem to be studied [3]. To inspect small defects, it is required that the inspection system has ultra-high resolution and sensitivity.

Different kinds of technologies, such as magnetic particle inspection, penetrant inspection, ultrasonic inspection, X-ray inspection, machine vision inspection and eddy current testing (ECT) can be employed for defects inspection [4,5,6]. Magnetic particle inspection and penetrant inspection methods are widely used NDT technologies. The visualization of the test results and the maturity of the testing system are their main advantages. However, for these technologies, it is difficult to realize automatic inspection [7,8,9]. X-ray detection methods have high accuracy and high inspection efficiency. The state-of-the-art X-ray inspection systems have the resolution at fine micron-level. The main drawbacks of an X-ray inspection system are: (i) the equipment is typically expensive and has large volume and (ii) it has radiological risk [10,11]. Acoustic microscopy is a kind of microscopy that employs very high-frequency ultrasound. Scanning acoustic microscopes (SAMs) can achieve resolutions in the micron range [12,13]. However, most acoustic inspection requires couplant and it requires careful preparation of the surface under test. Visual inspection with abundant processing algorithms is a popular surface inspection method, which has the advantages of being non-contact, fast and high-resolution. However, the influences such as shooting angle and lighting conditions in machine vision detection result in varying quality of the collected images, which affect the image processing and the accuracy of detection [14,15,16].

Eddy current testing (ECT) has the advantages of being non-contact, low-cost, fast and high resolution [17,18]. It is widely used to examine the structure of conductive materials to identify their structural integrity [19]. For ECT probes with a single pickup coil/sensor, a complex scanning path should be employed to cover an area, which makes the inspection inefficient [20]. Array probes with multiple receivers can obtain a C-scan image in a linear scan [21,22]. The incessant development of array probes has also led to an increase in detection accuracy. Machado et al. designed geometric array probes on a printed circuit board (PCB), which can detect small defects [23]. Liu et al. designed a null-offset planar eddy current sensor array to solve the problem of low resolution in the detection of buried defects in metallic materials [24]. Fan et al. improved the sensitivity of the array probe for crack identification by changing the space between the excitation coil and the sensing coil, and pasting the permalloy film on one side of the sensor [25]. Ma et al. combined the excitation of both windings with a multi-detection flexible array for crack detection [26]. Yusa et al. designed a uniform eddy current arrayed probe consisting of a large rectangular parallelepiped excitation coil and arrayed pancake pick-up coils [27]. Array ECT probes that are composed of magnetically coupled pancake coils and work with a transmit-receive configuration were widely used for plate and cylinder structure inspection [28]. A flexible probe with in-plane differential coils was proposed by Ye et al. The sensitivity of the probe for defects in different directions was increased by fusing images [29]. However, all the current probes have limited spatial resolution because of the restriction of the size of the pick-up coils. To detect small defects, the spatial resolution of the output images needs to be enhanced.

Identifying defects from the testing images utilizing proper algorithms is another important aspect of ECT. A continuous wavelet transform (CWT) and adaptive thresholding algorithms were employed simultaneously in [28]. An adaptive second-generation wavelet transform was utilized in [30] to reduce the noise. Buck et al. used artificial neural network (ANN) with the statistical technique of principal component analysis (PCA) to improve the defect detection performance [31]. Postolache et al. utilized ANN, including competitive neural network and multilayer perceptron to improve the accuracy of defect classification [32]. Morabito et al. studied fuzzy inference and fuzzy curves with a simplified set of features [33]. Gao et al. modeled low-rank and sparse decompositions in an alternating manner. By combining low-rank information into the sparse matrix, small defects were recognized more effectively [34]. Tao et al. preprocessed ECT image with spatial domain filtering and gradient feature edge extraction and then used a deep neural network-based target detection algorithm called mask-region convolutional neural network (Mask-RCNN) [35].

This paper presents an array probe, which enhances the image resolution by interpolating data from different rows of sensors. In addition, a patch-image model is utilized to suppress the noise and an image fusion method based on discrete wavelet transforms is employed to further improve the image quality. The rest of the paper is organized as follows: Section 2 introduces the design and operating principle of the probe. Then, Section 3 presents the experiment setup. Next, Section 4 presents the image processing algorithm and the results. Finally, Section 5 discusses the conclusion and future work of the study.

## 2. Probe Design and Operating Principle

### 2.1. Layout of The Coils

**Figure 1:**
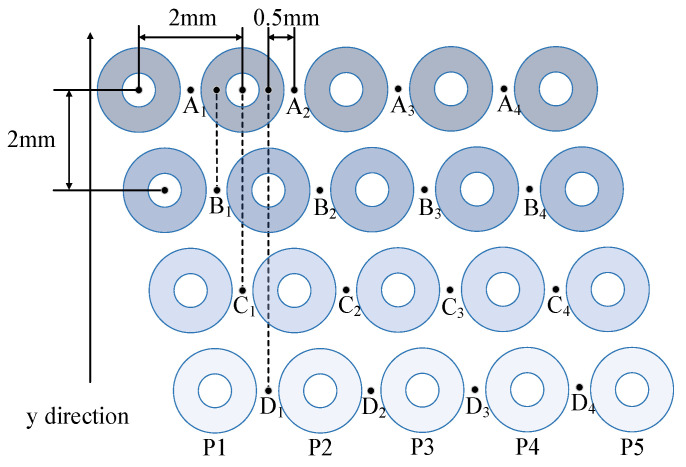
Coils layout of the resolution enhanced probe.

The layout of the coils in the probe is shown in Figure 1. The inner and outer diameters of the coils are 0.5 mm and 1.6 mm, respectively. The height of the coils is 1 mm and the diameter of the copper wire used to wind the coils is 0.04 mm. The number of turns of the coils is 240. The distance between the centers of each two adjacent coils in a row is 2 mm, and the space between every two adjacent rows is 2 mm. At each inspection moment, two adjacent coils in a row operate simultaneously. They are configured as a pair of in-plane differential coils to suppress the background signal and common mode noises [36]. The midpoint of the line connecting the centers of the two coils is defined as the sensing point. If there is only one row of coils, the resolution of the measurement along the horizontal direction (the distance between the sensing points) is 2 mm. The other three rows of coils are used to improve the image resolution. To this aim, the adjacent two rows of coils are offset by 0.5 mm along the horizontal direction. It is assumed that points A_1_–D_1_ are the sensing points of the first two coils of the four rows, respectively, and point A_2_ is the second sensing point of the first row. It is seen that points B_1_, C_1_, and D_1_ lag behind point A_1_ by 0.5 mm, 1 mm and 1.5 mm, respectively, along the horizontal direction if the points A_1_, B_1_, C_1_, D_1_, …, A_4_, B_4_, C_4_, D_4_. are aligned and plotted in a line. During the signal processing, the image resolution is increased from 2 mm to 0.5 mm. It is worth noting that this study is only to demonstrate the feasibility of the principle, so there are only five coils in one row. The number of coils in one row can be extended according to the requirement of the application.

### 2.2. Circuit Design

**Figure 2:**
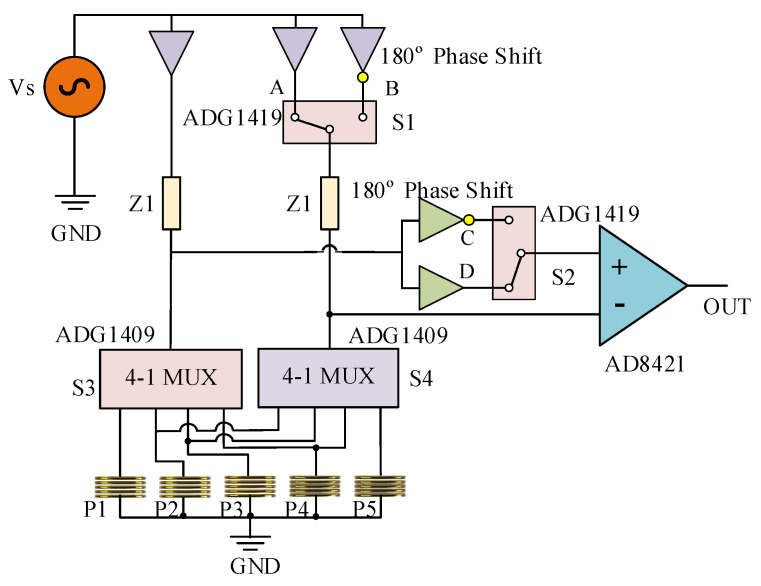
Circuit diagram of the probe for one row of coils.

The circuit schematic of the probe for one row is shown in Figure 2, where P1-P5 indicates the coils in the row. The Z1 is constant impedances. The bridge circuit is driven by an alternation voltage source Vs. The gain of the differential amplifier (AD8421) is 11.

At each time, two coils are selected to operate simultaneously by the two 4-to-1 multiplexers (ADG1409). The currents in the two coils can be configured to have the same direction (SD mode) or opposite direction (OD mode). When the two coils are operating in SD mode, switch S1 is connected to point A and switch S2 is connected to point D. When the two coils are operating in OD mode, switch S1 is connected to point B and switch S2 is connected to point C, in which case the current in the two coils has opposite flowing directions. The multiplexers are controlled by a data acquisition unit (NI PXIe5753), which is a digitizer adapter module with digital outputs. The time chart of the switches control is depicted in Table 1.

**Figure 3:**
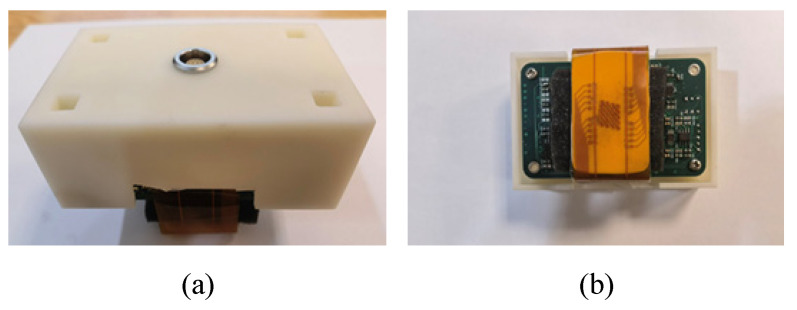
Photographs of the prototype probe: (**a**) top view and (**b**) bottom view.

The coils are attached to a flexible printed circuit board (FPCB) and then connected to the circuit board. The coils and the circuit board are packaged in a 3D printed enclosure. Photographs of the prototype probe are presented in Figure 3.

### 2.3. Model-Based Principle Study

A three-dimensional (3D) finite element method (FEM) model is built to study the operating principle of the proposed probe and predict the output images. In this paper, we used Python and Matlab to produce the simulation data, and all images of simulation results were drawn by Matlab. It is assumed that the materials have linear electromagnetic properties and are spatially and temporally non-dispersive. As the excitation frequency is low, the displacement current is ignored. The sizes of the coils are kept the same as the probe design. The dimensions of the conductive sample are length 10 mm, width 10 mm and depth 3 mm. The conductivity, relative permeability and permittivity of the conductive sample are $1.33\times 10^{6}\;S/m$, 1 and 1, respectively. The lift-off distance between the bottom of the probe and the top surface of the conductive sample is 0.5 mm. The frequency is 600 kHz and the excitation current density is $10^{9}\;A/m^{2}$. The governing equation of the FEM model is the reduced magnetic vector potential formulation [37].

(1) $$\nabla \times \nu \nabla \times A_{r}+j\omega \sigma A_{r}+\sigma \nabla V=\nabla \times H_{s}-\nabla \times \nu_{r}H_{s}-j\omega \sigma A_{s}$$

(2) $$\nabla \cdot (j\omega \sigma A_{r}+\sigma \nabla V)=-\nabla \cdot j\omega \sigma A_{s}$$

where ν and $\nu_{r}$ are the reluctivity and relative reluctivity of the material, respectively, j represents the imaginary unit, ω is the angular frequency and V is the electric scalar potential. $A_{s}$ and $A_{r}$ are the magnetic vector potentials due to excitation currents and induced currents, respectively. $H_{s}$ and $H_{r}$ are the source and induced components of magnetic intensity, respectively.

The change of the impedance of a coil in the probe due to the induced eddy current is written as (3), where n is the number of turns of the coil and I is the current flowing through the coil.

(3) $$\Delta Z=j\omega \sum_{i=0}^{n}\int A_{r}\cdot dl/I$$

Considering the circuit shown in Figure 2, the output voltage of the probe is written as (4) (+ for SD mode and – for OD mode). Here, G is the gain of the circuit and $V_{S}$ is the voltage of the excitation voltage source.

(4) $$V_{out}=\pm GV_{S}\left(\frac{Z_{0}+\Delta Z_{S3}}{Z_{1}+Z_{0}+\Delta Z_{S3}}-\frac{Z_{0}+\Delta Z_{S4}}{Z_{1}+Z_{0}+\Delta Z_{S4}}\right)$$

**Figure 4:**
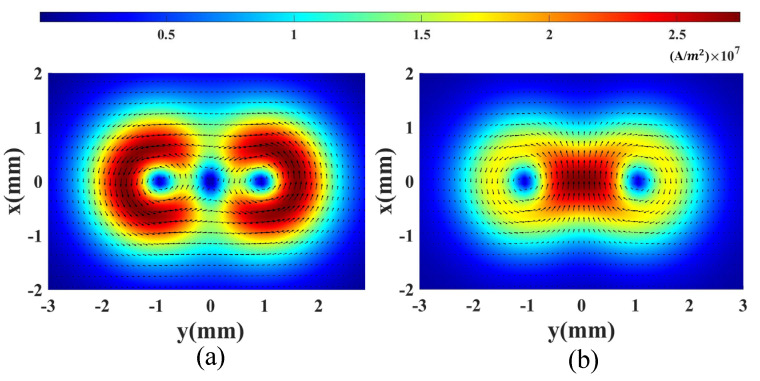
Induced eddy current in a defect-free conductive sample with (**a**) SD and (**b**) OD excitation.

The induced eddy currents in a defect-free sample 0.1 mm below the top surface with SD excitation and OD excitation are shown in Figure 4, where the color map presents the amplitude of the induced current and the arrows show the current flow direction. The induced current density at the middle point of the coils with SD excitation is about zero; however, the induced current density of OD excitation is the strongest at the middle point. It is seen that the distributions of the induced currents are different for SD and OD excitations.

**Figure 5:**
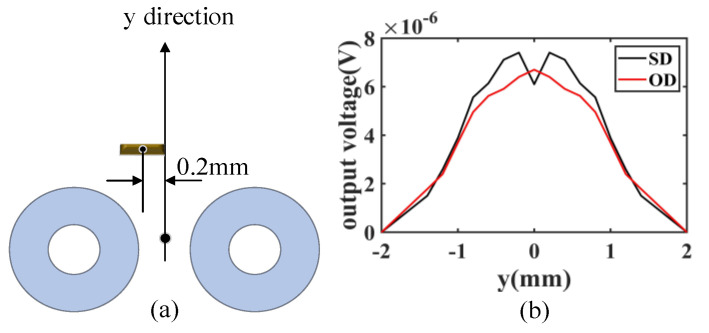
(**a**) The positions of the horizontal defect to the two coils in the simulation model, (**b**) the signal amplitude of the simulation.

The output signals of the coils are numerically calculated using the FEM model. A small defect with dimensions of 0.4 mm (length) × 0.2 mm (width) × 0.2 mm (depth) is simulated. The center of the defect is 0.2 mm shifted from the center of the two coils along the horizontal direction, as shown in Figure 5a. As the two coils are operating with a differential setting, if the defects are symmetrically related to the y–axis symmetry line of the two coils, the output will be zero. It should be noted that the probe is sensitive to a defect that is located anywhere under the probe, as it has multiple arrows of coils. The coils scan along the y-axis with a step size of 0.2 mm. The amplitudes of the simulation signals are shown in Figure 5b. The results show that the output voltages of the SD and OD modes are different for the same defect. This is because the distributions of the induced currents of the two modes are different, as shown in Figure 4, which means that more features of a defect can be extracted by obtaining the signals of the two models simultaneously in an inspection. In addition, the fusion of images of different modes can suppress random noise to a certain extent. Consequently, if the signals of SD and OD modes are collected in an inspection, it is possible to highlight the defect indication by fusing the results during the post-processing with a proper algorithm.

**Figure 6:**
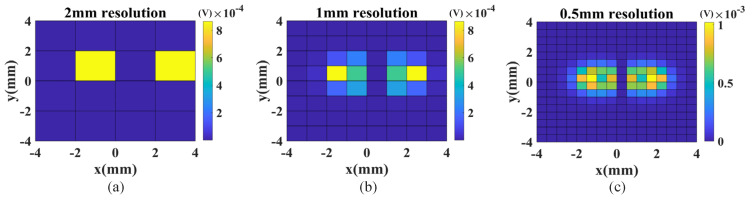
Simulation results: images of the horizontal defect with different spatial resolutions: (**a**) 2 mm, (**b**) 1 mm and (**c**) 0.5 mm.

Spatial resolution is an important factor that affects defect detection. To study the impression of the spatial resolution, images with different resolutions are generated using the FEM model. The dimensions of the defect are 1 mm (length) × 0.1 mm (width) × 0.1 mm (depth). The coils operate in the SD mode and scan over the horizontal defect point by point. The image resolution is changed by varying the scan step size from 0.5 mm to 2 mm. The images are shown in Figure 6. Compared to the image with resolution 2 mm (Figure 6a), the figure with resolution 0.5 mm (Figure 6c) shows the defect in much more detail, which is beneficial for defect detection and quantification. Therefore, there is a strong need to increase the image resolution.

## 3. Experiment Setup

**Figure 7:**
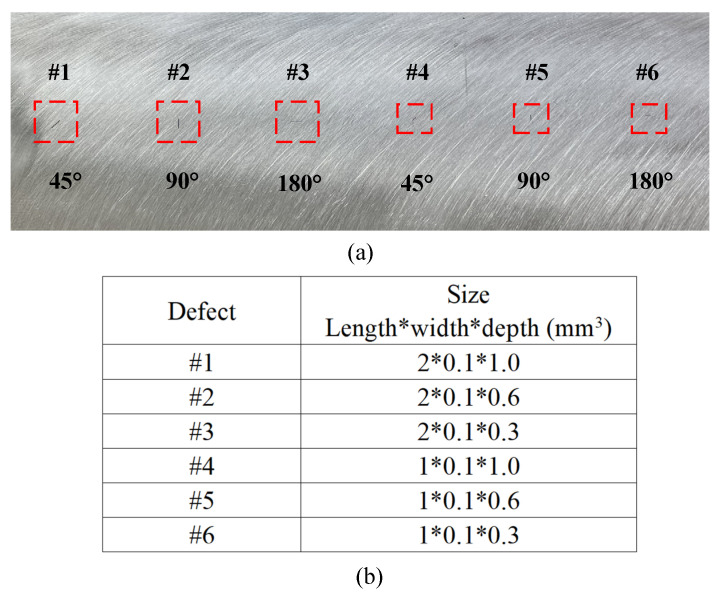
(**a**) Top view of the stainless sample with machined defects, (**b**) dimensions of the defects.

The probe is fixed to the gantry scanning system. The output signal of the probe is connected to and digitalized by the data acquisition unit (NI PXIe5753). The NI PXIe5753 also contains a signal generator card, which generates a sinusoid waveform. A 304 stainless-steel sample with machined defects is fabricated and tested by the probe. A picture of the sample is shown in Figure 7a, and the dimensions of the defects are specified in Figure 7b. It is worth noting that the dimensions of these defects are small.

### Raw Images

**Figure 8:**
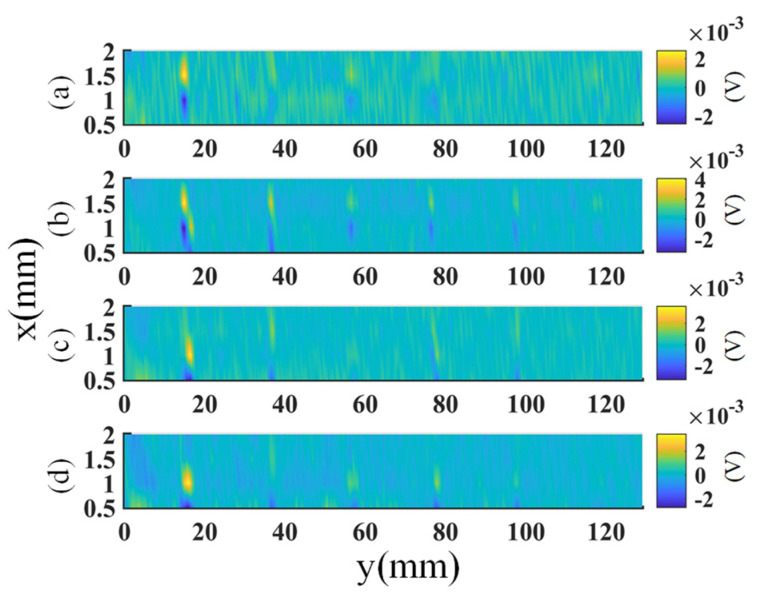
The raw images generated by the four rows of coils with SD excitation: (**a**–**d**) are the in-phase components of the outputs of the first row to the last row of coils, respectively.

The amplitude and frequency of the excitation voltage were 3 V and 600 kHz, respectively, which were chosen by optimization of the signal quality. The scan step size was set to 0.5 mm, and the speed was 10 mm/s. The probe is controlled to operate in SD and OD modes during the experiment. The raw images of the sample generated by the probe in SD mode are shown in Figure 8. Here, the in-phase component of the signal is plotted. The images of the OD mode are similar to the figures shown in Figure 8. Therefore, they are not presented to save space.

## 4. Data Enhancement Algorithms

**Figure 9:**
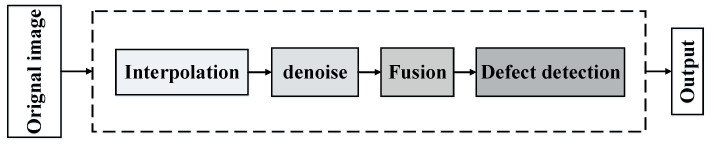
Flowchart of the data enhancement algorithms.

The flowchart of the data enhancement algorithms is presented in Figure 9. It includes image interpolation, denoise, fusion and defect detection.

**Figure 10:**
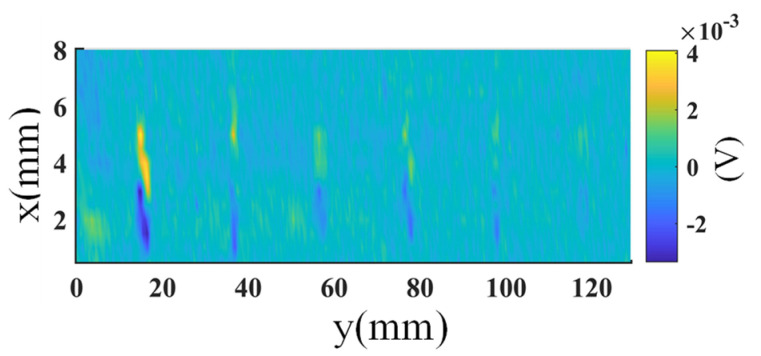
The resolution enhanced image is interpolated from the four images shown in Figure 8.

The images of the four rows of coils are interpolated to generate a resolution-enhanced image. The interpolation process is shown, as follows. As shown in Figure 1, each row has four measurement points. Assuming $\Gamma_{A}$, $\Gamma_{B}$, $\Gamma_{C}$ and $\Gamma_{D}$ are the data matrixes of the four rows, $\Gamma_{A}$ is written as (5). $\Gamma_{B}$, $\Gamma_{C}$ and $\Gamma_{D}$ are similar matrixes.

(5) $$\Gamma_{A}=\left[\begin{array}{ccc} A_{1}^{1} & \cdots & A_{4}^{1}\\ \vdots & \cdots & \vdots \\ A_{1}^{m} & \cdots & A_{4}^{m} \end{array}\right]$$

where m is the number of data points in a scan. Since the distance between every two adjacent rows is 2 mm and the scan step size is 0.5 mm, $\Gamma_{B}$, $\Gamma_{C}$ and $\Gamma_{D}$ are shifted along the scan direction accordingly and then merged together with $\Gamma_{A}$ to obtain the interpolated image $\Gamma_{i}$, as shown in (6). The interpolated image is presented in Figure 10. Intuitively, it is seen that the resolution enhanced image has better defect indications, from which all the defects can be detected. Following this, the resolution enhanced images will be analyzed.

(6) $$\Gamma_{i}=\left[\begin{array}{ccccccccc} A_{1}^{1} & B_{1}^{5} & C_{1}^{9} & D_{1}^{13} & A_{2}^{1} & B_{2}^{5} & C_{2}^{9} & \cdots & D_{4}^{13}\\ A_{1}^{2} & B_{1}^{6} & C_{1}^{10} & D_{1}^{14} & A_{2}^{2} & B_{2}^{6} & C_{2}^{10} & \cdots & D_{4}^{14}\\ & & & & \vdots & & & & \\ A_{1}^{m-12} & & & & \cdots & & & & D_{4}^{m} \end{array}\right]$$

**Figure 11:**
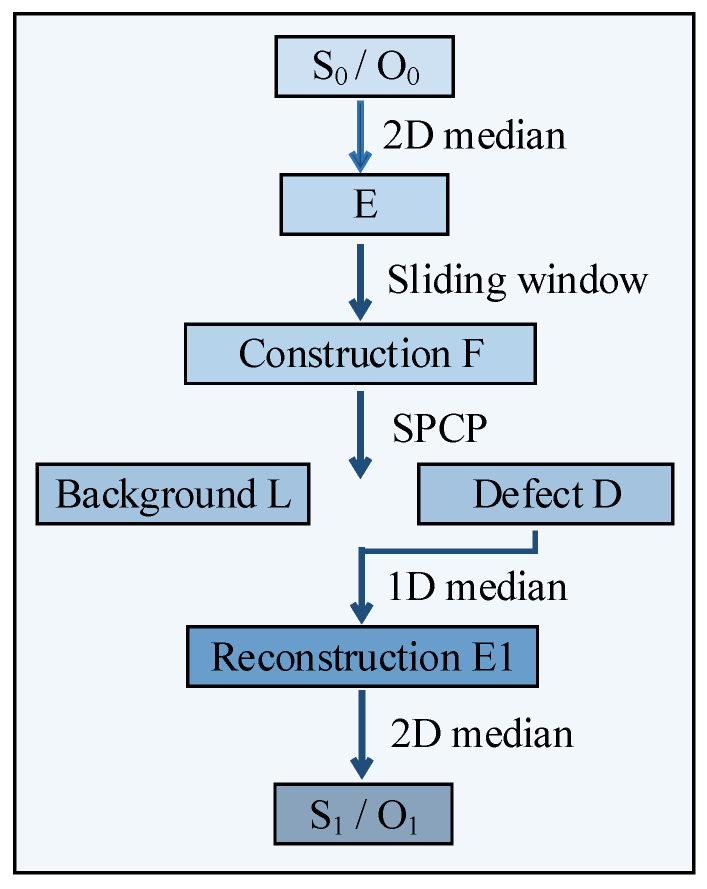
Flowchart of the denoise.

Noise is inevitable in the experiment, which may affect the small defects identification. Therefore, the images should be processed to suppress the noise. Using the Stable Principal Component Pursuit (SPCP) algorithm by constructing local patches [38] can suppress most noise. Figure 11 shows the flowchart of denoise. The raw images of the SD mode (S_0_) and OD mode (O_0_) are processed separately. Before using patch-image, a 2D median is applied to the raw image to reduce noise. Then, a series of patch-image blocks are obtained using a sliding window from the top left to the bottom right of the images, the sizes of which are 10 pixels × 10 pixels, and the step is 3. The construction matrix E is obtained by changing the position of the sliding window to F. Assuming the noise is independent and identically distributed, the patch-image model conforms to (7).

(7) E=L+D+N

where L is a low-rank matrix, D is a sparse matrix including defect information and N is the noise matrix. We assume ${\left\|N\right\|}_{F}\leq \delta$, for some δ>0. The problem is solved via SPCP to solve the following convex optimization problem, where λ is a positive weight constant [39].

(8) $$\min_{L,D}{\left\|L\right\|}_{*}+\lambda {\left\|D\right\|}_{1}\;\;s.t.\;{\left\|E-L-D\right\|}_{F}\leq \delta$$

**Figure 12:**
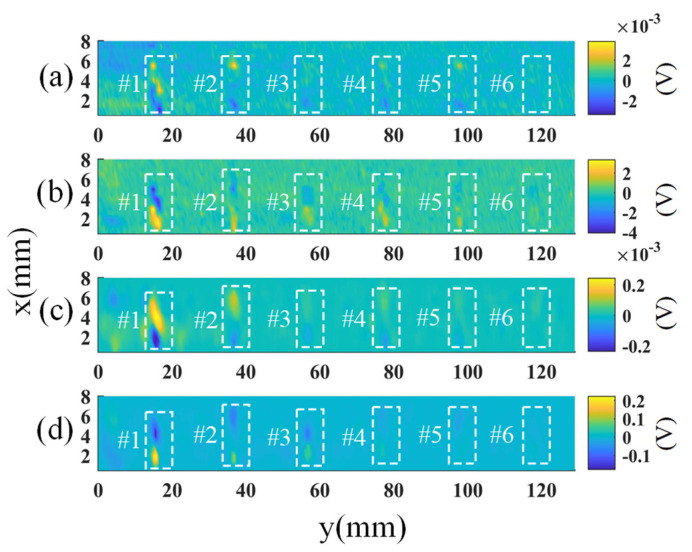
(**a**,**b**) are the original images of the SD and OD mode, respectively, and (**c**,**d**) are the corresponding processed images.

The optimization problem which is shown in (8) can be solved with the Accelerated Proximal Gradient (APG) [40]. The defect patch-image D and the background patch-image L are calculated. Then, the reconstructed image E1 is obtained by using the 1D median filter for a series of values obtained from overlapped image patches. Next, a 2D median filter is applied to E1 to suppress the noise. The output images are S_1_ and O_1_ for the SD and OD modes, respectively. Figure 12 presents the original images of the SD and OD modes (a,b) and the processed images (c,d). Compared with the original images, the noise is significantly suppressed in the processed images and the defect signals are more obvious, especially for the last three defects. Taking defect #5 as an example, its signal-to-noise ratio has increased from 7.69 dB to 28.99 dB. Here, the Signal to Noise Ratio (SNR) is calculated according to the ratio of the defect #5 signal amplitude compared to the noise signal amplitude that is not in the defect area.

**Figure 13:**
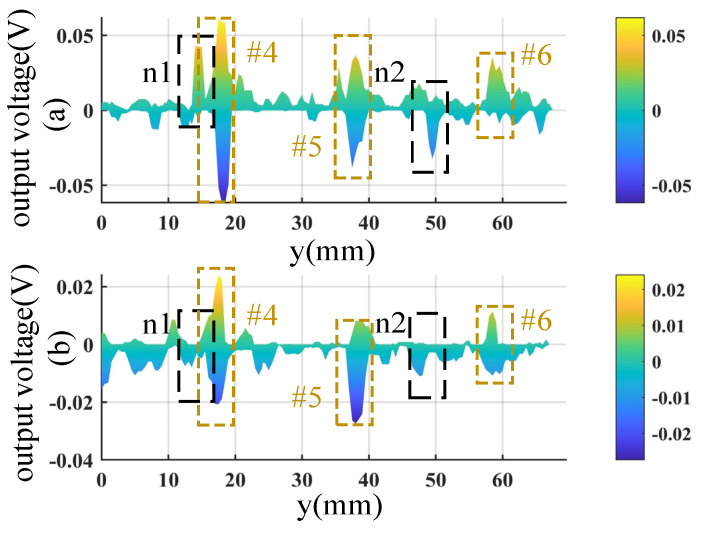
Zoom-in plots of defects #4−#6: (**a**) image of SD mode and (**b**) image of OD mode.

It is seen from Figure 12 that the indications of defects #4-#6 are weak. To show these defects more clearly, the zoom-in plots are presented in Figure 13. It is seen that all the defects have indications in the figures. However, there are some noises, some of which have a similar magnitude as the small defects’ signal, such as the noises n1 and n2 shown in Figure 13. Fortunately, the noise signals are not presented in the images of different modes consistently. For example, the noise n1 appears in the OD mode image, but it is not significant in the SD mode image. Similarly, n2 is presented in the SD mode image but not in the OD mode image. Meanwhile, the defects’ signals are presented in both modes. Given this characteristic, an image fusion method is employed to further suppress the noise and highlight the indications of the small defects.

**Figure 14:**
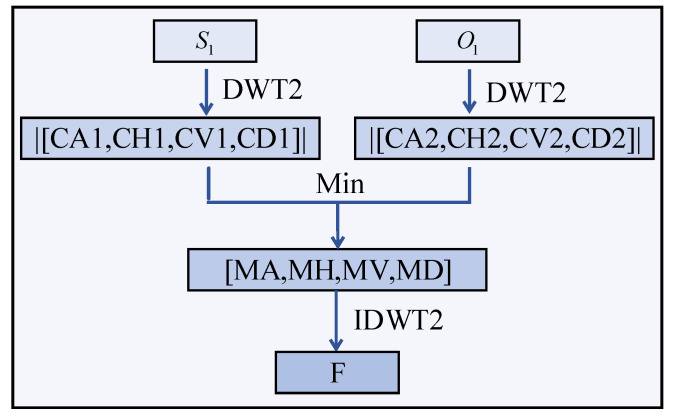
Flowchart of the image fusion.

Discrete wavelet transforms are utilized for image fusion [41,42]. The flowchart of the algorithm is shown in Figure 14. The input images are the preprocessed figures, namely S_1_ and O_1._ The image fusion algorithm is written as (9). First, the Mallat algorithm is employed to decompose the input images into four components, including low frequency (CA), horizontal high frequency (CH), vertical high frequency (CV) and diagonal high frequency (CD). Next, the absolute value of each component is minimized. Finally, the fused wavelet coefficients are inverted using an inverse wavelet transform to produce the final fused image F.

(9) $$F=\omega^{-1}(\min(\left|\omega (S_{1})\right|,\left|\omega (O_{1})\right|))$$

where ω is a wavelet transform and $\omega^{-1}$ is an inverse wavelet transform [43].

**Figure 15:**
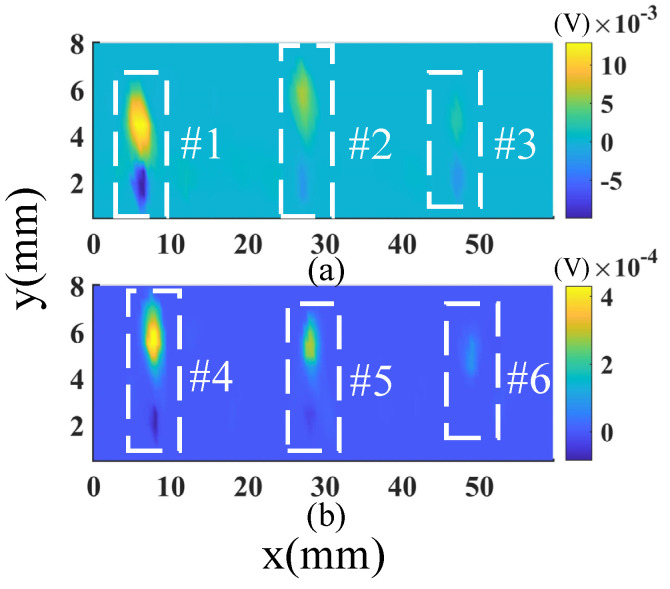
(**a**,**b**) are the SD mode fused images of defects #1−#3 and defects #4−#6, respectively.

The fused images of the defects are drawn in Figure 15. Here, the images of defects #1-#3 and #4-#6 are plotted separately to show the defects’ signal more clearly. It is seen that the noise is further suppressed by the image fusion. If a proper threshold value is applied, for example $1\times 10^{-3}$ V, which is one-tenth of the maximum defect signal, all the defects can be reliably detected from the fused image. The signal-to-noise ratio of defect #5 is further improved from 28.99 dB to 50.10 dB. It is worth noting that the defects are very small; for instance, the dimensions of defect #6 are only 1 mm (length) × 0.1 mm (width) × 0.3 mm (depth). It is challenging for conventional array ECT probes to detect such a small defect. The excellent defect indications in the fused image demonstrate that the method developed in this paper has super sensitivity for small defects inspection.

**Figure 16:**
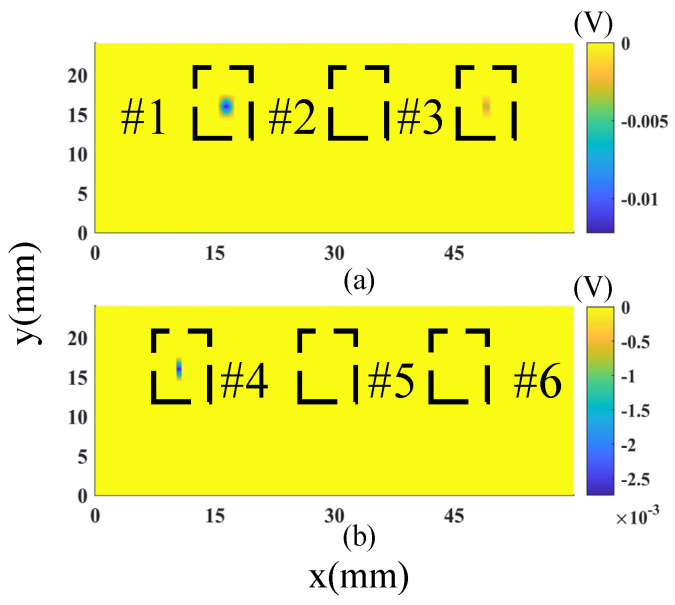
Horizontal channel images of the reference probe: (**a**) defects #1−#3 and (**b**) defects #4−#6.

The proposed probe is compared with a state-of-the-art ECT probe [44]. The reference probe consists of two rows of coils. The inner and outer diameters of the coils are 0.5 mm and 2.6 mm, respectively. The probe works in transmit–receive mode, e.g., at each time, a coil carrying AC current is activated as a transmitter, and three other coils are selected as receivers picking up the magnetic field. Due to the different positions of the pick-up coils related to the excitation coil, two channel images, namely vertical channel and horizontal channel, are obtained by the reference probe in an inspection, which is more sensitive to vertical defect and horizontal defects, respectively. The same sample was tested by the probe and the excitation frequency was kept identical to the previous experiment (600 kHz). The horizontal channel images of the reference probe after signal processing are shown in Figure 16. As the horizontal channel images of the reference probe are not as sensitive to the vertical defects as they are to the horizontal defects, it is reasonable that the defects #2 and #5 cannot be recognized from the images. It is worth noting that defects #1, #3 and #4 can be identified from the image, but defect #6 cannot be detected by the probe. As a comparison, defect #6 can be detected by the proposed probe, as shown in Figure 15, which proves that the proposed probe has better detectability for the defects.

## 5. Conclusions

This paper presents a resolution enhanced ECT array probe without sacrificing sensitivity. The outputs of the four rows are aligned and interpolated in a line during the signal processing, and in this way the image resolution is increased to 0.5 mm. Two coils of the probe operate simultaneously and are configured as in-plane differential measurements at the same time, so as to suppress the background signal and common mode noises. Simulation results showed that the probe has a similar sensitivity for horizontal defects and demonstrated the necessity of increasing the spatial resolution. Experimental results showed that the resolution enhanced image has better defect indications. A patch-image model and an image fusion method based on discrete wavelet transforms were utilized to enhance the contrast and remove noise interference. All the defects, including a small defect with dimensions as small as length × width × depth = 1 mm × 0.1 mm × 0.3 mm, can be detected, which demonstrates that the method developed in this paper has super sensitivity for small defects. This probe can be widely used for small defects inspection in the industry to maintain structural reliability and safety. Prior to field applications, more extensive optimization and testing should be conducted, such as: (i) the parameters of the probe, including the number of coils, geometry dimensions of the probe and operating frequency, etc., should be optimized according to the specified applications; (ii) the defect identification and quantification algorithm are yet to be further explored and tested; and (iii) the performance of the probe for different kinds of materials and robustness of operating in harsh industrial environments should be evaluated.

## Figures and Tables

**Table 1:** Chart of switching the multiplexers.

Time Slot	S1	S2	S3	S4	Mode
1	A	D	P1	P2	SD
2	B	C	P1	P2	OD
3	A	D	P2	P3	SD
4	B	C	P2	P3	OD
5	A	D	P3	P4	SD
6	B	C	P3	P4	OD
7	A	D	P4	P5	SD
8	B	C	P4	P5	OD

## Data Availability

The data presented in this study are available on request from the corresponding author.
